# A Systematic Review of Durum Wheat: Enhancing Production Systems by Exploring Genotype, Environment, and Management (G × E × M) Synergies

**DOI:** 10.3389/fpls.2020.568657

**Published:** 2020-10-29

**Authors:** Brian L. Beres, Elham Rahmani, John M. Clarke, Patricio Grassini, Curtis J. Pozniak, Charles M. Geddes, Kenton D. Porker, William E. May, Joel K. Ransom

**Affiliations:** ^1^Agriculture and Agri-Food Canada, Lethbridge Research and Development Centre, Lethbridge, AB, Canada; ^2^Crop Development Centre and Department of Plant Sciences, University of Saskatchewan, Saskatoon, SK, Canada; ^3^Department of Agronomy and Horticulture, University of Nebraska, Lincoln, NE, United States; ^4^South Australia Research and Development Institute, Adelaide, SA, Australia; ^5^Agriculture and Agri-Food Canada, Indian Head Research Station, Saskatchewan, SK, Canada; ^6^Department of Plant Sciences, North Dakota State University, Fargo, ND, United States

**Keywords:** durum wheat, genotype, environment, management, G × E × M, agronomy

## Abstract

According to the UN-FAO, agricultural production must increase by 50% by 2050 to meet global demand for food. This goal can be accomplished, in part, by the development of improved cultivars coupled with modern best management practices. Overall, wheat production on farms will have to increase significantly to meet future demand, and in the face of a changing climate that poses risk to even current rates of production. Durum wheat [*Triticum turgidum* L. ssp. durum (Desf.)] is used largely for pasta, couscous and bulgur production. Durum producers face a range of factors spanning abiotic (frost damage, drought, and sprouting) and biotic (weed, disease, and insect pests) stresses that impact yields and quality specifications desired by export market end-users. Serious biotic threats include Fusarium head blight (FHB) and weed pest pressures, which have increased as a result of herbicide resistance. While genetic progress for yield and quality is on pace with common wheat (*Triticum aestivum* L.), development of resistant durum cultivars to FHB is still lagging. Thus, successful biotic and abiotic threat mitigation are ideal case studies in Genotype (G) × Environment (E) × Management (M) interactions where superior cultivars (G) are grown in at-risk regions (E) and require unique approaches to management (M) for sustainable durum production. Transformational approaches to research are needed in order for agronomists, breeders and durum producers to overcome production constraints. Designing robust agronomic systems for durum demands scientific creativity and foresight based on a deep understanding of constitutive components and their innumerable interactions with each other and the environment. This encompasses development of durum production systems that suit specific agro-ecozones and close the yield gap between genetic potential and on-farm achieved yield. Advances in individual technologies (e.g., genetic improvements, new pesticides, seeding technologies) are of little benefit until they are melded into resilient G × E × M systems that will flourish in the field under unpredictable conditions of prairie farmlands. We explore how recent genetic progress and selected management innovations can lead to a resilient and transformative durum production system.

## Introduction

An estimate by the UN-FAO indicates that, by 2050, the global demand for agricultural products will have risen by 50%. Meeting this demand will require traditional development of improved cultivars coupled with modern best management practices as well as innovations that are transformational. Achievement of this goal on existing cropland will require a significant increase in rates of genetic gain in grain yield for crops such as wheat (*Triticum aestivum* L.), increasing the current rate of gain (ca. 1% p.a.) by 30–40% (FAO, 2017; Cassman and Grassini, 2020). Climate change will be an added challenge to productivity improvement (Reynolds et al., 2016). Durum wheat (*Triticum turgidum* L. var durum Desf.) is the 10th most important and commonly cultivated cereal worldwide with a yearly production average of 40 million tonnes (MT) (2016/17). Typically, durum wheat production represents 5% of total wheat production with a planting area of 16 M hectares globally (International Grains Council [IGC], 2020). Wheat and wheat products could account for 20% of protein and calories consumption per capita for a global population of 9.7 billion in 2050 (CRP-WHEAT, 2016). Durum is produced primarily for making pasta, but is also an important ingredient for couscous and bulgur, particularly in North Africa and the Middle East. These products use durum semolina resulting from milling of the hard-textured durum wheat kernel. In some countries such as Italy, regulatory standards specify that pasta must be made with 100% durum semolina (Sopiwnyk, 2018). The production of pasta requires grain with high protein content, gluten strength, and high yellow pigment content (resulting largely from lutein), which provides the characteristic yellowness that is expected from the pasta. Only a few regions in the world are capable of producing durum that meets the high standards for end-use suitability. In this review article, we will discuss the current state of durum production and explore how recent genetic progress and selected management innovations can lead to a resilient and transformative durum production system.

### Statistics and Regional Specific Summaries (Canada, Australia, and the United States)

Durum wheat is grown in many of the same countries that produce common wheat, with Italy as an important producer (4.95 MT) within Europa, along with the Commonwealth of Independent States (CIS), North America, South America, Asia, Africa, Oceania, and Turkey (3.62 MT) (International Grains Council [IGC], 2020). A majority of the world’s durum wheat is planted in North America, with Canadian production typically around 7.8 MT, which is almost three times the production of the United States (US) and Mexico (International Grains Council [IGC], 2020). In Canada, southern Saskatchewan is the largest wheat durum producer, supplying 81 percent of the total Canadian durum wheat produced from 1990 to 2017. Canada’s durum export volume has increased from 2.7 to 4.5 MT from 1990 to 2017, which underscores the significant contribution of durum production to the Canadian export market (Statistics Canada, 2019). Canada now leads the world in durum wheat exports, with about half of all durum wheat available for export grown in Canada. Mexico and the EU contribute 17 and 16%, respectively. The remaining 17% is exported from other countries including the United States, Australia, Mexico, and Kazakhstan. From the period 2013–2014 to 2017–2018, Italy, Algeria, Morocco, United States, and Japan were the top five importing countries for Canadian durum wheat (International Grains Council [IGC], 2020).

In Australia, durum wheat represents a relatively small component of the Australian wheat crop. Durum production is largely confined to rainfed production in southeastern Australia (South Australia, Victoria, and New South Wales and a small part of southern Queensland) and small pockets of irrigated production. Wheat production in eastern Australia has averaged ∼16 MT per annum over the last 10 years (ABARES 2017), whereas durum production averages ∼4,00,000 tonnes (T) but has fluctuated substantially between ∼50,000 and 8,00,000 t over the last decade (Kniep, 2008; Ranieri et al., 2012); and represents on average 3% of the eastern Australia wheat crop. Australian durum wheat production is relatively stable, and the annual supply of grain is split equally between domestic consumption and export markets, which is somewhat dependent on seasonal fluctuations (Ranieri, 2015).

In the United States, durum is produced primarily in two regions, the desert southwest region under irrigated regimes, and in the central region of the northern Great Plains under rainfed conditions. The largest area planted to durum is in North Dakota (Table 1), followed by Montana, Arizona, and California. There is also minor production in Idaho (irrigated), South Dakota and Minnesota. In the last two decades, there has been a substantial reduction in the area planted to durum in the United States. Most of this can be attributed to reductions in North Dakota, where there has been a reduction of nearly 800,000 hectares over the period. Farmers in North Dakota have opted to grow other crops due to the challenges of meeting the high quality standards to reach the top grade and the lack of financial incentives relative to other crops including hard red spring wheat, which has somewhat less stringent quality requirements. Lastly, as with other regions, the lack of genetic resistance to Fusarium head blight (FHB) caused by *Fusarium graminearum* Schwabe [telomorph: Gibberella zeae Schwein (Petch)] has shifted hectarage away from durum to wheat crops with higher levels of resistance.

**TABLE 1:** Durum production trends in the United States over the last 20 years (USDA National Agricultural Statistic Service database).

**State**	**Area planted in 2018**	**Average annual growth or reduction in area planted since 1998^†^**	**Average yield over of the period**	**Average annual yield increase since 1998^‡^**
	(ha)	(ha year^–1^)	(*t* ha^–1^)	(*t* ha^–1^ year^–1^)
Arizona	28,745	801	6.74	0.031
California	16,194	−338	6.74	−0.037
Montana	340,081	5,176	1.85	0.021
North Dakota	445,344	−39,734	2.12	0.040

*^†^Derived from the linear regression of hectares planted, 1998 through 2018. ^‡^Derived from the linear regression of average statewide yield, 1998 through 2018.*

#### Market Access

A recent challenge for producers is slumping durum exports to Italy, the world’s largest pasta maker. As reported in April 2018, despite the high exceptional quality of Canadian durum and as Italy’s biggest durum supplier, the company Barilla has cut back its imports from Canada by 35%. Italy has expressed concerns over Maximum Residue Limits (MRLs) for glyphosate in Canadian durum as the reason for the blockade. In the northern Great Plains, where durum is managed within no-tillage, soil conservation system, glyphosate is widely used as a pre-plant application to control weeds. Glyphosate applied to durum after it reaches physiological maturity is also approved as a pre-harvest weed control in the United States and Canada. It is most commonly used when environmental conditions prolong the process of drying after maturity. Glyphosate applied prior to harvest has been found to only marginally hastens the rate of dry down in fully mature wheat (Darwent et al., 1994), but it does an excellent job of controlling any green tillers or weeds that might produce seed and increase dockage at grain terminals. Although the MRL is five parts per million for wheat according to Health Canada’s current guidelines, the accepted limit of glyphosate established by the Italian pasta industry is under 10 parts per billion. Exporters argue the limits are too low because glyphosate is already used within acceptable limits of the herbicide in the grain production system.

The expanded use of glyphosate as a preharvest aid may be associated with the expanded use of fungicides for FHB control, as plants treated with this late fungicide application tend to remain green longer as the leaves and stems are not affected by late-season diseases that might naturally desiccate them. Glyphosate is highly mobile in the plant and moves to areas of high metabolism. When applied prior to maturity, it can accumulate in developing kernels in sufficient quantity that it can affect germination and therefore is not currently approved for use in seed production or on barley intended for malt (Jenks et al., 2019). Even when applied after physiological maturity, however, it is possible to detect traceable amounts of glyphosate in the grain (Cessna et al., 1994). When applied according to the manufacturer’s label, glyphosate does not affect milling and baking characteristic of spring wheat (Manthey et al., 2004) or the functional quality of durum (Zollinger et al., 1999). From a toxicological standpoint, glyphosate is considered to be one of the safest herbicides available, as its mode of action is directed towards an enzyme not found in mammals. Most countries consider pesticide residues in their food based on the guidelines found in the Codex Alimentarius which has a permitted residues limit of 30 ppm of glyphosate in wheat. Some countries like Japan and Canada have lower permitted limits of 5 ppm. Nevertheless, after the International Agency for Research on Cancer concluded that glyphosate may be linked to cancer, many end users have become concerned with any detected levels of glyphosate in the grain. There are alternative chemicals registered for pre-harvest weed control in durum, but they are primarily effective on broadleaf weeds and would have minimal impact on the desiccation of the durum crop (Jenks et al., 2019). Reverting to the practice of swathing the crop and leaving the cut grain in windrows to dry before combining may also be challenging. This practice is labor-intensive and increases operational input costs. Grain quality is also at greater risk as there is less potential airflow permeating through the windrow such that, if there is a rain event, there is increased risk of quality downgrading from weathering and sprouting.

### Yield Constraints and Emerging Issues for Durum Wheat Production

#### Attainable Yield and the Global Yield Gap Atlas

**FIGURE 1:**
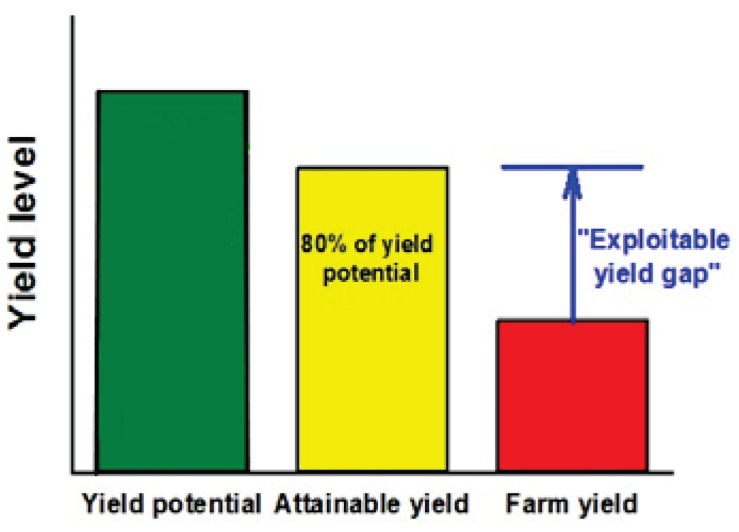
Representation of yield potential, attainable yield, farm yield, and yield gap (Van Ittersum et al., 2013). Appropriate permissions have been obtained from the copyright holder(s) of this work.

Our ability to fully harness the genetic yield potential of newly deployed genetics and traits in the latest durum cultivars is determined by site-specific parameters and local management practices. Achieving food security and protecting carbon-rich and biodiverse natural ecosystems from massive conversion to cropland ultimately depend on our ability to sustainably increase crop yields on currently cultivated land (Cassman, 1999). Until recently, for most of the world, including data-rich regions such as the United States Corn (*Zea mays* L) Belt and Europe, there were no reliable data on yield potential—the maximum attainable yield as determined by climate and soil in the absence of nutrient deficiencies and biotic stresses (Evans and Fischer, 1999). To help meet this need for data and help producers achieve these potential yield gains, researchers from University of Nebraska-Lincoln (United States) and Wageningen University (WU) began development of the Global Yield Gap Atlas (GYGA) (GYGA,^1^) in 2011, with the goal of defining regions’ exploitable yield gaps (Figure 1). In this figure, yield potential is determined by temperature, precipitation, solar radiation, carbon dioxide, and, in the case of rainfed crops, also by water supply and soil properties influencing soil water balance (Evans, 1993; Van Ittersum et al., 2013). The attainable yield is about 80% of the yield potential (Cassman et al., 2003; Lobell et al., 2009). Achieving yield gain above the attainable yield is difficult as the extra investment on inputs and labor is not cost effective. The exploitable yield gap represents the difference between average on-farm or actual yield and the attainable yield.

The GYGA provides a web-based platform for estimating yield gaps that is transparent, accessible, reproducible, geospatially explicit, agronomically robust, and applied in a consistent manner throughout the world. A standard protocol for assessing yield gaps was developed by leading scientists, which is based on a strong focus on understanding the local farming system and making use of the best available data sources (Grassini et al., 2015; Van Bussel et al., 2015). A number of studies have been published on these topics using the GYGA approach (Hochman et al., 2013; Merlos et al., 2015; Van Oort et al., 2015; Espe et al., 2016; Marin et al., 2016; Van Ittersum et al., 2016; Timsina et al., 2018). Currently, wheat yield gaps have been developed for the EU, AUS, South America, and India; however, yield gaps for Canada and the United States have yet to be developed by GYGA and remain unknown. A project to obtain estimates for Canada and the United States in a joint collaborative project utilizing GYGA methodology was initiated in 2019. In GYGA, yield potential is simulated based on long-term weather data, local soil, cropping system data, current crop sequences, and dominant management practices such as sowing date, plant density, and cultivar maturity (Grassini et al., 2015).

An alternative approach was used to investigate wheat yield trends, attainable yields and yield gaps for the 10 largest wheat producing countries in the world and more localized yield statistics at the state or county level. These data were assembled from available government sources. Attainable yield was determined using an upper quadrant analysis to define the upper frontier or yields over the period of record and yield gaps calculated as the difference between attainable yield and actual yield for each year. In all countries, the attainable yield increase over time was larger than the yield trend indicating the technological advances in genetics and agronomic practices were increasing attainable yield. Average wheat yield gap using this method report that Australia is about 24%, spring wheat in Canada (Saskatchewan and Alberta) is between 21 and 24%. France, Germany, Mexico, and United States are approximately 14, 9, 10, and 12%, respectively. Observations across all the wheat production regions shows no apparent trend in closing the yield gap. The average yield gap using this method is a reflection in the variation in weather among growing season and management of soil water followed by enhanced agronomy. A series of challenges exist as we attempt to assemble regional data with much finer resolution data to be able to elucidate specific soil differences and weather/climate data. The country level data is useful for looking at trends but is not robust enough for managing yield gap solutions as an accurate measure of the yield gap due to management. Therefore, more country partners with finer scale data are needed to make progress on the quantification of yield gaps, the factors which constrain growers achieving water limited yields, and potential strategies for reducing the yield gap with true G × E × M synergies (Hatfield and Beres, 2019). For example, the most recent study on yield gaps of wheat in Australia conducted in-silico experiments to determine the impact on grain yield of sub-optimal practices and tested these against emerging best practices as developed by agronomists (Hochman and Horan, 2018). With this approach, it was possible to quantify reasons for the yield gap. Average national losses were due to growers not applying enough nitrogen, failure to adopt conservation tillage techniques, suboptimal weed control during summer fallow, low seed densities, and delayed sowing. Moreover, the GYGA atlas determined that the yield gap that is related to management for Australia is around 50%. Irrespective of the method to define yield gaps, progress toward closure in the future will require local producers to adopt practices that increase their climate resilience in wheat production systems (Hatfield and Beres, 2019).

**FIGURE 2:**
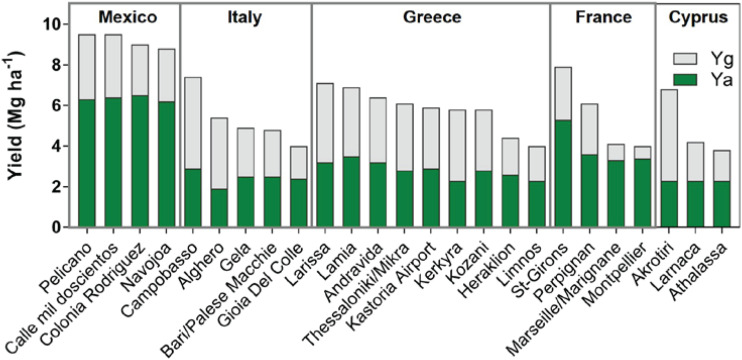
Yield potential for durum wheat in different producing countries. The green and gray portions of the bar correspond to the actual durum yield (Ya) and the yield gap (Yg), respectively. Wheat was rainfed in all cases except for Mexico, where it was managed with irrigation. Source: Global Yield Gap Atlas. Original work.

Specific to durum yield gaps and for the purposes of this paper, the GYGA methodology was utilized recently along with existing data to calculate durum yield gaps for specific durum producing regions (Figure 2). France and Mexico represent regions with the smallest yield gap, with some regions in France nearly achieving yield gap closure. The higher yield potential in Mexico is a reflection of production under irrigation, which can largely eliminate the limitation caused by insufficient water supply from precipitation and stored soil moisture at sowing. Regions within Italy, Greece, and Cyprus with the highest yield potential also displayed actual yields that were not measurably different from the lowest yield potential regions, which creates a wide yield gap exceeding 50%. This suggests large room at the farm level to improve yield *via* improved agronomic management.

#### Global Challenges for Yield Attainment – Biotic Threats

While insect threats such as wheat stem sawfly *Cephus cinctus* Norton (*Hymenoptera: Cephidae*) and orange blossom wheat midge (*Sitodiplosis mosellana*) can be major production constraints, their cyclical nature includes years to a decade where their presence is below economic thresholds. Fungal diseases, however, and most notably FHB caused by *Fusarium graminearum*, have had the greatest impact on the durum industry in the Canadian Prairies and the Great Plains of United States as well as other wheat-growing regions (Beres et al., 2018). Durum is also particularly vulnerable to crown rot (*Fusarium pseudograminearum, Fp*), tan spot (*Drechslera tritici-repentis*), Septoria leaf blotch (*Mycosphaerella graminicola*), bacterial leaf streak (*Xanthomonas translucens*), leaf rust (*Puccinia triticina*), and stem rust (*Puccinia graminis*), and stripe rust or yellow rust (*Puccinia striiformis*) which causes the most damage when pustules develop early on the flag leaf and disrupt photosynthesis (Ransom et al., 2017). While many diseases can potentially reduce the yield and quality of durum, integrated management strategies exist that can help reduce the effects of these diseases. However, genetic progress to develop cultivars resistant to diseases lags behind other wheat classes in both North America (Ransom et al., 2017) and Australia (Hollaway et al., 2013). In Australia, durum cultivars with a high level of resistance to *Fp* have not been identified. This has reduced durum production to farming systems with lower levels of crown rot. Farming practices such as retaining more cereal residue and increasing the intensity of wheat rotations can increase the amount of inoculum in the soil, which can further increase the likelihood of infection (Summerell et al., 1989). With key durum growing regions of Australia predicted to become hotter and drier, yield losses due to crown rot disease are expected to increase. While the development of varieties with improved crown rot resistance has been shown to reduce inoculum levels in soils, improved management solutions must focus on limited exposure to water stress as a key G × E × M strategy to reduce yield loss from crown rot in Australia. One proposed strategy is the development of durum cultivars with improved frost tolerance and subsequently amenable to earlier planting, which would minimize exposure to terminal drought and water stress during grain fill.

Weeds are one of the largest contributors to wheat yield loss. Weed interference can result in spring wheat yield loss ranging from 5% to greater than 80% (Harker et al., 2017). Currently, a paucity of information is available highlighting the magnitude of weed-induced yield loss in durum compared with other wheat classes. However, the information that is available suggests that durum and bread wheat may have similar tolerance to weed interference. One exception showed greater grain yield loss induced by increasing densities of wild oat (*Avena fatua* L.) in a lath-house experiment in a bread wheat cultivar compared with a durum wheat cultivar; suggesting that durum wheat could have greater tolerance to weed interference (Henson and Jordan, 1982). In their study, densities of wild oat equivalent to 162, 406, and 812 plants m^–2^ reduced grain yield of durum wheat on average by 34, 53, and 67%, respectively, when durum wheat was sown at a density equivalent to 162 plants m^–2^. A crop rotation study in a rainfed Mediterranean environment of central Italy concluded that weed control and nitrogen supply are among the most important factors impacting durum wheat yield and grain quality, and that these factors are of greater importance in years with excess rainfall and low temperatures during reproductive development of durum wheat in organic production systems (Campiglia et al., 2015).

Although the use of herbicides has enabled the adoption of conservation cropping systems, the increased reliance on herbicides for weed control has led to the evolution of herbicide resistance (Walsh and Powles, 2007). It has been estimated that overall, the total cost of weeds for growers with resistance can increase by as much as $55 ha^–1^ higher than those without resistance (Llewellyn et al., 2016). The major weed constraint to durum wheat production in Australia is annual ryegrass (*Lolium rigidum* L.). In South Australia, annual ryegrass has evolved considerable resistance to all post-emergent herbicides used in cereal crops (Boutsalis et al., 2012). Similarly in Canada, a recurring theme around weed dynamics in durum fields is the occurrence of herbicide-resistant biotypes of these species (Heap, 2019). In Alberta and Saskatchewan, Canada, separate green foxtail (*Setaria viridis* L.) populations have been found with resistance to acetyl-CoA carboxylase (ACCase)-inhibiting (group A/1), acetolactate synthase (ALS)-inhibiting (group B/2) and microtubule-inhibiting (group K1/3) herbicides. Wild oat populations with resistance to ACCase inhibitors, ALS inhibitors, or lipid synthesis (group N/8) inhibitors are also present in these areas. The increasing frequency of multiple herbicide-resistant wild oats in the Canadian prairies has raised concern over effective management of these populations in cereal and pulse crops. In Canadian durum, no post-emergence herbicide options exist currently for management of wild oat populations with multiple resistance to both the ACCase and ALS inhibitors, assuming cross-resistance to all active ingredients within this herbicide modes-of-action. This has increased grower reliance on pre-emergence herbicide options.

#### Global Challenges for Yield Attainment – Abiotic Threats

Abiotic stress is a phenomenon encountered in all durum production regions, but Australia is particularly vulnerable as it appears most likely to first experience climate change that will challenge yield and quality targets for durum. Agronomic and genetic solutions have been successful in mitigating yield lost to some degree but challenges remain; therefore, a considerable research effort is still required. Durum yield losses in Australia are primarily due to water stress (Liu et al., 2015) similar to well-published studies in wheat from lack of rainfall during spring, which causes a mild water deficit stress prior to anthesis, moderate stress at anthesis and becomes more severe during grain fill (French and Schultz, 1984). Breeding for genotypes adapted to pre- and post-anthesis water-deficit stress has been a major target of Australian breeders (Liu et al., 2015) and will need to remain a significant priority (Alahmad et al., 2019) given that durum production is in Mediterranean areas most likely influenced by climate change, and wheat yields in Australia are beginning to stall (Hochman et al., 2017).

Frost events in spring are also common, which causes yield loss by directly reducing the number of grains via sterility induced by the combined effect of cold, desiccation and freezing damage to the floral organs and developing grain (Al-Khatib and Paulsen, 1984; Boer et al., 1993; Fuller et al., 2007; Zheng et al., 2015). A large effort has been undertaken in Australia to screen for improved frost tolerance; however, Australia durum cultivars have a significantly higher susceptibility to frost damage than bread wheat and barley (*Hordeum vulgare* L.) (Cocks and Cullis, 2019). Equally high temperatures and heat shock during sensitive reproductive growth stages and grain fill can also result in a yield penalty (Gomez-Macpherson and Richards, 1995). A key study by Zubaidi et al. (2000) concluded the critical issue with durum wheat in South Australia was not its yield potential per se, but its ability to maintain yield when challenged by environmental stress. Agronomic solutions to maintain yield and reduce potential exposure to water stress, heat and frost can be achieved by manipulating plant development and sowing date (Bassu et al., 2009). A series of experiments in South Australia compared the relative performance of durum and bread wheat when exposed to the same level of abiotic stresses. When flowering at a similar time the yield gap ranged from 0.6 to 1.5 t/ha due to increased sterility from increased sensitivity of durum to abiotic stresses such as heat and reproductive frost (McCallum et al., 2019). The best strategy available to growers under these conditions is to ensure that flowering occurs during the optimum flowering period (Flohr et al., 2017) when the combined stresses of frost, drought and heat risk (Flohr et al., 2017) are minimized. A more recent environmental constraint, and a further example of potential negative effects of climate change, is the fact that autumn rainfall required to establish crops in Australia has diminished (Cai et al., 2012). Increased effort is therefore needed to develop genetics with diversity in crop phenology patterns to ensure growers can respond and sow earlier or later than currently practiced, and still achieve flower on time in order to overcome the yield decline (Kirkegaard and Hunt, 2010).

## Designing a Resilient Durum Cropping System

### Improvements to the “G” in the G × E × M Paradigm in Canada

**FIGURE 3:**
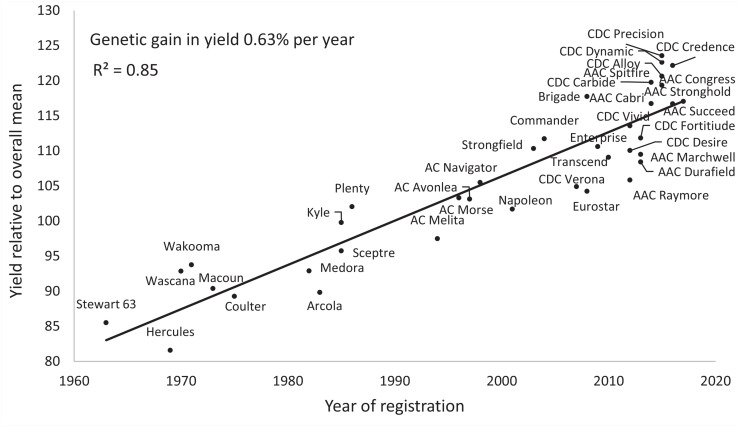
Durum wheat genetic gain of grain yield from 1963 to 2017 in Canada. Source: Prairie Recommending Committee for Wheat, Rye, and Triticale (http://pgdc.ca/committees_wrt_pd.html). Appropriate permissions have been obtained from the copyright holder(s) of this work.

The rate of genetic gain in yield of durum wheat in Canada averaged 0.63% (approximately 21.5 kg ha^–1^) per year from 1963 to 2017 (Figure 3). There was an increase in the number of new cultivars brought into production during the last decade, reflecting the major increase in funding for breeding programs, principally by farmers. De Vita et al. (2007) reported a gain of 19.9 kg ha^–1^ year^–1^ for durum yield in Italy from 1900 to 1990. Royo et al. (2007) reported a rate of gain of 0.36 to 0.44% per year in a historic set of Italian and Spanish durum cultivars. Investigation of rates of gain in a more recent period (1980–2009) in Spain showed a similar gain of 0.44% per year up to 2003, with little change thereafter (Chairi et al., 2018). Declining rates of genetic yield gain, much less doubling current rates, could be a concern in future, and widening the genetic diversity of crossing programs is necessary, and is a priority of breeding programs unless new genetic diversity is discovered.

Retrospective studies of the factors underlying genetic improvement in grain yield identified key contributing traits. For example, increased harvest index (the ratio of grain weight to total plant biomass) has contributed to yield improvement in durum (De Vita et al., 2007) and bread wheat (Beche et al., 2014). This is largely associated with the introduction of semi-dwarf cultivars, now grown exclusively in many countries, although less so in the northern Plains area of North America, particularly in the case of durum. Future increases in harvest index are unlikely because it is impractical to further shorten straw length due to issues of mechanical harvest, so yield increases must come from improved overall biomass, coupled with resistance to lodging. An exhaustive long list of possible selection targets for potential yield and methods for their selection, have accumulated (Fischer and Rebetzke, 2018). Yield increases over time were associated with improvements in plant physiological processes such as higher stomatal conductance (leading to lower leaf canopy temperature) and photosynthetic rates (Fischer et al., 1998; De Vita et al., 2007; Beche et al., 2014) and associated traits such as leaf chlorophyll content (Beche et al., 2014). Manipulation of factors related to photosynthetic efficiency might contribute significantly to future yield gains (Martin et al., 2011), but the probability of success and associated timeline are highly uncertain. Up to the present, physiological traits were improved indirectly through selection for grain yield because direct measurement of such traits was limited by the technology available to measure them. However, research suggests co-selection of physiological traits with yield *per se* can maximize genetic gain, and the availability of new tools associated with ground-based or aerial platforms is driving new interest in assessing crop canopy traits such as transpiration, chlorophyll content, and leaf area, in addition to plant phenology, in high throughput phenotyping systems (White et al., 2012). This can now be done at the scale required for breeding programs (Araus and Cairns, 2014). Combining these data with genomic selection, made possible by next-generation sequencing and SNP genotyping technologies, might help increase the rate of genetic gain compared to traditional breeding approaches (Rutkoski et al., 2016; Crain et al., 2018).

Resistance to disease continues to be a major factor in the maintenance or improvement of durum wheat yields. Leaf rust, yellow or stripe rust and stem rust are globally important biotic constraints to wheat production (Eversmeyer and Kramer, 2000). Other leaf diseases such as tan spot (caused by *Pyrenophora tritici-repentis*) and Stagonospora nodorum blotch and *Septoria tritici* blotch limit yields, which can be mitigated by genetic resistance. For example, Fernandez et al. (2010) demonstrated that a 16% reduction in tan spot symptoms increased durum grain yield by 17%. FHB is a major disease of durum wheat, capable of causing yield reduction and loss of marketability of infected grain. Both genetic resistance and crop management can mitigate the effects and contribute to incremental increases in yield potential (Beres et al., 2018).

Going forward, the challenge is to vastly increase rates of genetic gain in wheat yield. The underlying key to achieving genetic gain for grain yield is having appropriate genetic variability from which to select. Past success is largely based on recombining alleles within elite germplasm and is now supported by advances in genomic selection (Haile et al., 2018; Montesinos-López et al., 2019) and high-throughput phenotyping (Condorelli et al., 2018). Hybrid wheat breeding is another approach to improving durum wheat, with indications of hybrids with 10% greater yield than the mid-parental value (Gowda et al., 2010). However, an effective pollination control system for hybrid seed production remains elusive. Thus, real progress will require an infusion of new genetic diversity into breeding programs. This can be done by crossing with related landraces or wild relatives, recognizing strategies to minimize linkage drag of undesirable traits will be required. Introgressions from wild relatives have been used successfully for disease resistance, although this has usually entailed many cycles of backcrossing and selection to develop agronomically suitable cultivars. New genomic technologies may be able to assist in the introgression process and shorten the time to cultivar release (Dempewolf et al., 2017), particularly if coupled with approaches to speed generation acceleration (Alahmad et al., 2018). Genome editing is a promising technology that will allow precise generation of new allelic variants for use in breeding. The clustered regularly interspersed short palindromic repeats (CRISPR/Cas9) system was demonstrated to increase grain size in durum and common wheat (Zhang et al., 2016). The CRISPR/Cas9 system requires precise determination of the desirable allele for mutation, which in turn requires accurate genome sequence annotation. Annotation of wild emmer (*Triticum dicoccoides*) and durum wheat genome assemblies will assist in this process (Avni et al., 2017; Maccaferri et al., 2019). However, routine application of gene editing requires extensive research to link gene annotation with phenotypic function (Barabaschi et al., 2016; Adamski et al., 2020).

The development of strong international collaborations during the last decade, such as the Wheat Initiative^2^ and associated projects such as the 10+ Wheat Genome project^3^ has significantly increased our understanding of the wheat genome. This knowledge paves the way for future research that will link genes to phenotypic function. Maccaferri et al. (2019) recently demonstrated the linkage for the example trait, grain cadmium concentration. Understanding multi-gene (quantitative) traits such as grain yield will be more challenging, but ultimately achievable.

### Examples of Innovations in the “M” of the G × E × M Paradigm

#### Seeding Systems – A Re-think on Sowing Density

The role of management is to limit or overcome losses due to abiotic and biotic factors with the aim to achieve the genetic potential for grain yield. A fundamental step is the consideration of planting density, which is often under-utilized in the context of optimizing genetic yield potential, imparting yield stability and improving crop uniformity and competitive ability. Decisions around optimal sowing density are often influenced by convenience, past practice, equipment, and the cost of purchasing seed of new cultivars. This decision-making process can lead to less than ideal seeding rates as seed input costs may be perceived as cost-prohibitive if yield potential is not achieved. Thus, despite increases in genetic yield potential, sowing densities during the 1970s through to the 1990s was fairly static at a rate of fewer than 200 seeds m^–2^ (Grant et al., 1974; Fowler, 1982; Entz and Fowler, 1991). However, research in wheat (Beres et al., 2010a,b, 2011) indicates higher rates are needed similar to experiences in other crops such as corn where increases in sowing density were needed before grain yield of corn hybrids could reach potential and eclipse older conventional corn cultivars (Duvick, 2005). In wheat, Beres et al. (2011) reported a positive linear improvement to grain yield in durum at rates as high as 450 seeds m^–2^, which was more than double the rate of standard practices at the time. The potential of higher seeding rates to exploit the yield of new durum genetics was confirmed in follow-up studies and is now the norm in western Canada (Beres et al., 2010b, 2011; Nilsen et al., 2016; Isidro-Sánchez et al., 2017; Ye et al., 2017).

The adoption of high seeding rates also reduces the potential for weed competition and can be an important tool for disease management as crop uniformity can influence the prevalence of some diseases (Beres et al., 2018). Variation in crop uniformity generally prolongs the flowering period of durum wheat for the most sensitive time for FHB infection (Buerstmayr et al., 2019), resulting in greater disease severity in the crop. Higher sowing densities in wheat reduces tillering and results in more main stems in a field, which will shorten flowering and days to maturity. Greater uniformity would also improve fungicide efficacy as crop staging to determine appropriate application windows would be facilitated greatly if the crop was less variable (Beres et al., 2018).

#### Seeding Systems – Seed Treatments for Abiotic Stress Resistance

Seed-applied fungicidal or insecticidal applications are traditionally based on mitigating biotic stress caused by insects or seed/soil-borne pathogens (Hewett and Griffiths, 1986; Mathre et al., 2001). McMullen and Stack (2009) observed a substantial improvement in germination and seedling vigor even when diseased seeds are used. Vernon et al. (2009) reported improved wheat stand with imidacloprid, in the presence of wireworms. Recently, the importance of seed treatments has been proposed not only in managing the biotic stressors but in mitigating abiotic stressors like heat stress, drought, wind desiccation, and frost, which usually arise from cold ambient and soil temperatures at planting and emergence.

A study of soybean (*Glycine max* L.) seed treated with thiamethoxam reported accelerated germination and larger seedlings concomitant with buffering against the negative effects of water deficit (Cataneo et al., 2010). A Canadian study on eastern Canadian spring wheat reported that a dual fungicide (difenoconazole and metalaxyl) and an insecticide (thiamethoxam) enhanced the freezing tolerance of seedlings (Larsen and Falk, 2013). Ford et al. (2010) established that neonicotinoids such as imidacloprid and clothianidin induce salicylic acid-associated responses, which elicit plant protection to pathogens such as powdery mildew concomitant with abiotic stress tolerance.

Recent work in wheat in western Canada with dual fungicide/insecticidal seed treatments have all reported improvements to crop stand establishment and grain yield in wheat production systems (Beres et al., 2016; Turkington et al., 2016; Ye et al., 2017). The same seed treatments also improved crop vigor and yield stability when integrated into a system with factors related to seed size and sowing density. The most notable responses occurred in the weakest agronomic system with thinner seeds and low seeding rates. An economic analysis supported that a dual (fungicide/insecticidal) seed treatment provides greater gross returns (CAN+$31 ha^–1^) as well as improved net returns (+$22 ha^–1^) (Beres et al., 2016). Moreover, comparisons between spring and winter growth habits indicate greater responses in winter wheat. This suggests the prolonged period of abiotic stress associated with winter growth habits can be mitigated effectively with seed treatments to overcome the negative aspects of the “E”.

#### Seeding Systems – “Ultra-Early” for Life Cycle Synchrony

Planting date and environment play an impactful role in durum wheat agronomic and end-use characteristics, regardless of cultivar grown (Forster et al., 2017). Historically, the recommended planting date for wheat on the Canadian Prairies has been early- to mid-May in southern regions and a targeted deadline of June 10 in the northern latitudes of the Parkland region. These dates were prescribed largely to meet crop insurance deadlines, or, are a product of on-farm logistics. For example, He et al. (2012) indicated that average commercial spring wheat planting dates were May 9, May 14, and May 15, for Swift Current, Saskatoon and Melfort, SK, respectively. These dates appear late when compared with earlier planting dates they predicted using a DSSAT-CSM model. This is largely to ensure the crop has developed sufficient biomass so as to capture maximum radiation by June 21 when photoperiod tends to peak on the Prairies. Early planting, therefore, is an important integrated crop management strategy designed to optimize genetics and to fully synchronize crop phenology with maximum solar radiation and environmental conditions that achieve high attainable grain yield and quality (Beres and Wang, 2018). Durum wheat yield losses in Australia are primarily due to water stress (Liu et al., 2015) similar to well-published studies in wheat from lack of rainfall during spring which causes a mild water deficit stress prior to anthesis, moderate stress at anthesis and becomes more severe during grain fill (French and Schultz, 1984). Agronomic solutions to maintain yield and reduce potential exposure to water stress, heat and frost can be achieved by manipulating plant development and sowing date (Bassu et al., 2009; Collier et al., 2020). To facilitate the timing of optimal flowering, growers need to match a genotype development speed with sowing date. An emerging trend in Australia has been the shift towards earlier planting systems to overcome the negative impacts of climate change (Hunt et al., 2019b). However, there is less variation in flowering time of the commercially available durum wheat cultivars relative to bread wheat in Australia, which is restricting a grower’s ability to adopt this strategy for durum production.

Forster et al. (2017) conducted a study on durum wheat in North Dakota, United States and concluded early planting improved grain yield regardless of cultivar or environmental conditions during vegetative and reproductive growth. Early planting helps to enhance soil moisture usage, which is an important factor for growing durum and can be profitable for the environments with low and high rainfall as well. They also concluded that quality diminishes in late plantings regarding semolina extraction, gluten index, and wet gluten values and a significant reduction in test weight and grain yield on different cultivars with delayed planting. Conversely, protein and kernel yellow pigment contents, vitreous kernels, and falling number were more related to cultivar and did not depend on planting date and environment.

Research in Canada has reported a number of benefits associated with an “ultra-early” planting regime whereby soil temperature in the top 5 cm of the soil surface dictates when planting should occur instead of a traditional calendar date. The authors report that grain yield was not compromised and often maximized when seeding occurred at around 2^*o*^C soil temperature. A greater reduction in grain yield was observed when planting was delayed until soil reach 10^*o*^C, despite extreme environmental conditions after initial seeding, including air temperatures as low as −10.2^*o*^C and as many as 37 nights with air temperatures below 0^*o*^C (Collier et al., 2020). An opportunity associated with such a system that results in earlier maturity is a refined integrated pest management strategy. For example, one major pest of durum wheat is the orange wheat blossom midge, which attacks wheat during anthesis. An ultra-early planting strategy using soil temperature as a trigger facilitates an asynchrony between the vulnerable host plant phenological stage and the primary window of pest infestation (Collier et al., 2020). The same management strategy is used by producers to mitigate FHB infection such that earlier flowering coincides with less optimal environment conditions for fungal infection. Early planting coupled with an early-maturity cultivar managed with practices that optimize crop uniformity might lessen the probability of FHB infection and reduce the risks associated with early fall frost events (Beres et al., 2018).

## Case Studies of G × E × M

**FIGURE 4:**
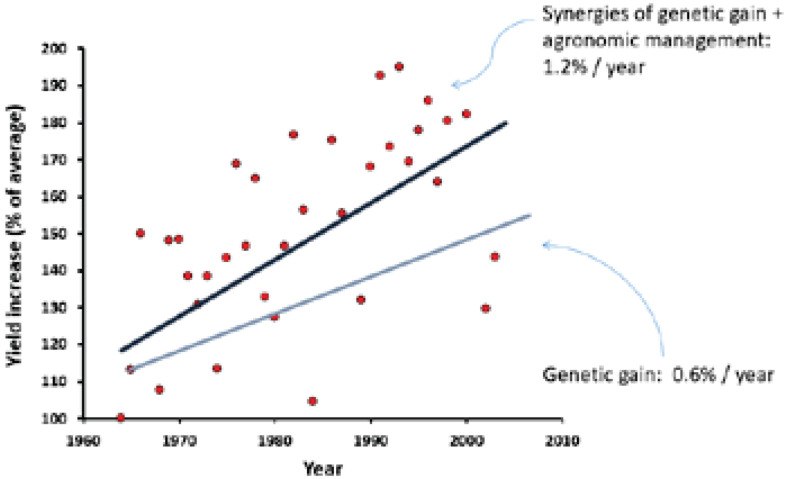
The role of agronomy management and genetics on yield increase over time (Clarke et al., 2010). Appropriate permissions have been obtained from the copyright holder(s) of this work.

Synergies of genetic gain and agronomic management are critically important in achieving higher yields, including the adoption of the new high-yield cultivars, applying precision farming, optimizing the nutrient application, zero tillage, appropriate seeding rates, and irrigation management. Overall, the interaction of genetics and agronomy management strategies has resulted in up to 1.2% increase in yield per year over time in Canada (Figure 4), approximately double of the rate of the genetic gains alone (Clarke et al., 2010). While there is no evidence yet that rates of genetic gain have begun to decline, sustaining current rates long-term, or further increasing rates of gain, will require new genetic diversity, breeding technologies, and strategies. To double overall production, assuming the current balance of genetic and agronomic improvement is maintained, the rate of genetic gain in grain yield will have to be increased to greater than 1% per year.

### Fusarium Head Blight Management – Experiences in Canada and the United States

Fusarium head blight is a devastating disease of wheat and a serious production and marketing constraint for durum production. It can cause significant yield losses concomitant with reductions to durum quality via development of mycotoxins in the seed if the fungal pathogen fully develops without intervention. The mycotoxin, deoxynivalenol or DON, is present in the grain and causes serious downgrades when delivered to market. Moreover, once DON levels exceed 2 ppm, markets that would accept the grain are limited. *Fusarium graminearum*, the causal organism of FHB, overwinters on infected residues of small grains and corn. Synchrony between the release of spores and flowering of the subsequent wheat crop phase ensures a high rate of infection within the spike/spikelet, leading to high rates of seed infection. The spread of conidia to the spike is facilitated with conditions that transport spores upward from the colonized stubble (splashing), and/or through release of ascospores into the air where windborne dispersal occurs to neighboring fields. The cycle perpetuates through the annual colonization of host tissues and when infected seed is planted without mitigation tactics. Conditions most favorable for the development of FHB are high humidity, frequent rainfall and relatively warm night temperatures at heading, especially in regions where host crop residues are present. Therefore, inoculum is rarely considered a limiting factor in the development of this disease. Currently, the recommendations for FHB control include growing durum after a broadleaf crop, using an approved fungicide at flowering, and grow resistant varieties if available. Though there are notable differences between varieties in their susceptibility to FHB, there are currently no varieties that are considered resistant or even moderately resistant (Beres et al., 2018).

**FIGURE 5:**
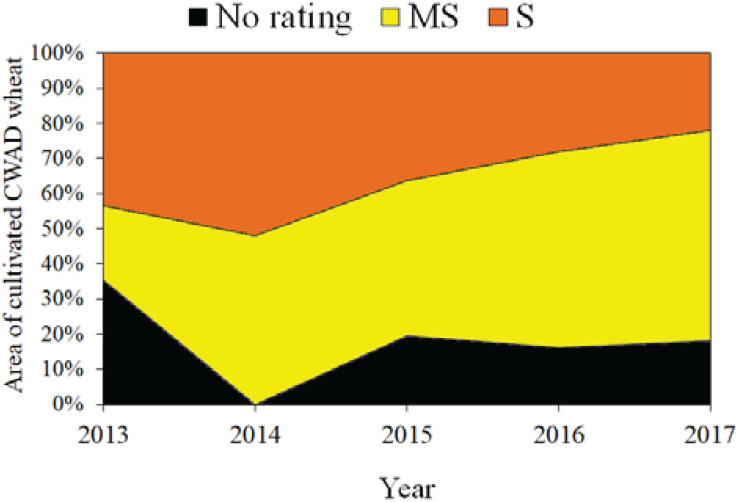
Durum cultivar adoption in the Canadian Prairies based on FHB rating. Original work.

Since 2000, there has been significant investment in breeding FHB resistant cultivars. In Canada, cultivars are rated along a resistance continuum as follows: “Susceptible” (S) (e.g., Strongfield), “Moderately Susceptible” (MS) (e.g., CDC Credence, Brigade, and Transcend), “Intermediate” (I), “Moderately Resistant” (MR), and “Resistant” (R). To date, the best cultivar rating remains at the level of “MS” (Beres et al., 2018). In the Canadian Prairies, Manitoba is the most vulnerable region due to frequent occurrences of FHB outbreaks; therefore, little to no durum is grown in that region. Some progress has occurred with respect to FHB resistance as MS cultivars have now been released. Since 2015, there has been an increase in the cultivated area of the MS varieties compared to other varieties. The rate of adoption of cultivars and respective ratings for FHB in the Canadian Prairies during 2013 to 2017 is presented in Figure 5. Producers have displaced susceptible cultivars with improved resistance, such that in 2017, 78% of cultivated area of durum wheat were planted with cultivars rated “MS” (Agriculture Financial Services Corporation [AFSC], 2020; Manitoba Agricultural Services Corporation [MASC], 2020; The Western Producer, 2020). Obviously, improvements to resistance that results in the release of I or MR cultivars is critically important to stabilize or expand both the production area and market access.

Given the lack of genetic control options, greater attention to management strategies is required, particularly when durum seed is sourced from a seed lot with greater than 10% Fusarium damaged kernels. Mitigation strategies are most successful when the disease cycle is interrupted at or prior to the flowering stage to prevent spore dispersal and/or host infection. While the main management strategy for FHB mostly focusses on in-crop fungicide applications to control FHB, seed-applied fungicides have been recognized as an effective method to improve yield and stand establishment when FHB stress exists (Ye et al., 2017). Durum wheat seed treatments that apply difenconazole, triticonazole, maneb, or fludioxonil have been found to significantly improve germination and reduce Fusarium seedling blight in field trials with 5–45 % levels of infection, but with no significant improvements in yield (Jørgensen et al., 2012). Other wheat trials have reported that seed treatments increased emergence and yield when levels of seed infection were high (>50%), with no improved emergence and grain yield in low levels of seed infection (≤10%), and increased emergence with no change in grain yield in moderate levels of infection (25–35%) (May et al., 2010).

The lack of genetic resistance in durum varieties to FHB and poor efficacy with management practices has led to a reduction of durum production in North Dakota and the migration of durum to the drier parts of the state. In order to improve efficacy of management practices, research was initiated in North Dakota that created a G × M context by exploring genetic aspects with the inclusion of both susceptible cultivars and a breeding line with some level of known resistance. Experiments were conducted in four environments in 2017 through 2019. These experiments consisted of five or six genotypes (released varieties and one advanced line) that were either treated with fungicide or not. Yield and DON levels were measured after harvest. The released varieties are commonly grown in North Dakota and their current rating of FHB tolerance is listed. The fungicide treatment consisted of applying prothioconazole plus tebuconazole when most of the spikes had reached 50% flowering (Table 2).

**TABLE 2:** Effect of fungicides on DON levels and yield in four environments in North Dakota, 2017 and 2018.

	**Carrington 2017**				**Carrington 2018**		**Prosper 2018**				**Prosper 2019**	
	**DON**		**Yield**		**DON**		**DON**		**Yield**		**DON**	
	**None**	**Fung.**	**None**	**Fung.**	**None**	**Fung.**	**None**	**Fung.**	**None**	**Fung.**	**None**	**Fung.**
	**(ppm)**		**(t ha^–1^)**		**(ppm)**		**(ppm)**		**(t ha^–1^)**		**(ppm)**	
Carpio (MS)^†^	6.7	0.8	3.57	4.88	0.7	0.6	3.3	4.1	3.46	3.95	9.0	9.0
D09555 (−)	6.4	0.5	3.50	5.71	1.7	0.7	3.2	3.0	3.49	3.80	11.1	8.6
Divide (MS)	3.9	0.6	3.63	4.68	2.4	0.9	3.2	3.5	3.76	3.75	10.5	4.9
Joppa (MS)	6.3	0.6	3.44	4.95	2.1	1.4	4.9	2.5	3.29	3.93	11.1	4.3
Mountrail (S)	6.2	0.9	3.19	4.36	2.0	1.0	2.6	3.3	3.50	3.80	15.7	5.0
Tioga (MS/S)	6.4	0.4	3.78	4.93	0.6	1.0	3.8	2.4	3.72	4.07		
Mean	6.0	0.6	3.52	4.92	1.6	0.9	3.5	3.1	3.54	3.88	11.5	6.4
Fung.^‡^	**^†^		**		*		NS		**		*	
Variety	**		**		**		**		**		*	
V × F	**		0.06		**		0.08		NS		*	

^†^MS, moderately susceptible, S, susceptible. ^‡^Level of statistical significance of the factor within the row. * = 0.05, ** = 0.01, NS = not significant.

The response of genotype and fungicide on DON levels varied considerably between environments. In Carrington 2017, DON was affected by the interactions of wheat variety and fungicide (Table 2). This was due to the more susceptible genotypes having high levels of DON (more than 6 pm) when no fungicide was applied, and all genotypes having similarly low levels of DON (less than 1 ppm) when treated with fungicides. This location was in contrast to two environments where DON levels when averaged across genotypes, were reduced by only 0.4 to 0.6 ppm DON with the application of a fungicide but similar to the 2019 data. Genotypes differed significantly across all environments for DON levels. Within the released varieties, no one variety consistently had the lowest DON. Though there were significant interactions between variety and fungicide treatment, the moderately resistant varieties were still responsive to fungicides, showing the value of integrating control practices. The yield was only measured at two locations. Yield increased dramatically with the application of fungicide at the Carrington 2017 location, but only modestly at the Prosper location. Only at the location exhibiting the highest FHB infection was there a genotype by fungicide interaction for yield.

While fungicide-based options are the primary management response, other components should be considered. When designing crop rotations, the “1 in 4” principle is particularly important for FHB mitigation (Kutcher et al., 2011, 2013), whereby a susceptible crop phase occurs only once in every 4 crop years. Moreover, sequencing durum after a broadleaf crop offers advantages such as: potential increases in yield; reductions in disease pressure such as tan spot and Septoria; a reduction in inoculum load of *Fusarium graminearum*; preventative buildup of soil-borne pathogens; rotation of herbicide chemistry diversity, which helps delay the buildup of herbicide-resistant weeds (Knox, 2018). The need for uniformity has been discussed in the context of the influencing factors of seeding rate and seed treatments; however, crop nutrition such as appropriate levels of phosphorous contribute greatly to crop uniformity (Hooker et al., 2016).

Durum is particularly vulnerable to FHB infection when produced under irrigated conditions. However, alterations to irrigation management, particularly during pre- and post-anthesis, is an effective strategy for controlling FHB. Flowering is an important phenological stage for durum wheat, which typically begins 3 days after the head has fully emerged and lasts for about 3–5 days. This period also coincides with maximum water uptake requirements by the crop. It is recommended that irrigation should be terminated for 8–10 days during flowering to reduce humidity in the durum canopy. Reducing water availability during this critical phase of crop development could compromise yield potential. However, durum grown on loam or clay loam soils may tolerate 10 dry days without significantly impacting yield, if the 50–100 cm of soil in the root zone is at field capacity just before flowers emerge (McKenzie and Woods, 2018). Thus, intensification of irrigation management is required in order to mitigate FHB without compromising yield.

The over-arching principle for FHB management is the manipulation of agronomic factors that facilitate completion of critical crop developmental phases, such as flowering, while doing so rapidly and uniformly, as a consequence of early sowing and increased seeding rates. Experiences in Canada and the United States illustrate the importance of linking together multiple management factors. For example, a management strategy of foliar fungicide and/or ST+foliar fungicide generally produced higher yields with greater stability, particularly for susceptible cultivars in high FHB environments (Ye et al., 2017). These strategies and the adoption of practices involving proper fungicide selection, and optimal application timings and methods will lead to improved yield stability and quality in high risk environments (Beres et al., 2018; Newlands, 2018). This is critically important for durum as it would help to overcome the necessary use of moderately susceptible cultivars until such time that breeding can catch up and deploy genetics with improved levels of resistance.

### Integrated Approaches to Weed Management

Cultural weed management plays an important role in managing herbicide-resistant weeds. Durum cultivar selection and agronomic management (G × M) can improve the ability for durum to compete with weeds and thus reduce selection pressure for herbicide resistance. Several physiological traits are linked to the ability of wheat to compete with weeds, including early-season vigor, tillering, leaf area index, plant height, and allelopathic potential, among others. Fewer physiological traits impact the ability of durum wheat cultivars to compete with weeds compared with bread wheat cultivars (Zerner et al., 2008). This is likely due to differences in growth habit among durum and bread wheat. In general, durum cultivars exhibit reduced tillering and leaf area index; two traits that are often associated with early canopy closure. The ability for durum to compete with weeds is associated closely with cultivar height, where taller cultivars are generally more competitive (Zerner et al., 2008; Beres et al., 2010b; Giambalvo et al., 2010). For example, Zerner et al. (2008) studied how the height of near-isogenic wheat lines (NILs) contributed to the ability for wheat to compete with oats (*Avena sativa* L.). In their study, tall bread wheat and durum wheat NILs reduced oat seed production by 26 and 41%, respectively. Similarly, a study of three durum wheat cultivars in the presence and absence of a surrogate weed [barley (*Hordeum vulgare* L.)] showed differences in the ability to compete with weeds among the durum cultivars (Giambalvo et al., 2010). In their study, the ability for durum wheat to compete with the surrogate weed was associated with the stature of the cultivar (i.e., taller plant stature contributed to greater ability to compete with weeds) and the nutrient uptake ability of the cultivar, which reduces nutrients available to competing weeds. Much less is known about allelopathy of durum wheat compared with bread wheat, and the contribution of allelopathy to genotypic differences in the ability for durum wheat to suppress weeds is a current knowledge gap (Oueslati, 2003; Fragasso et al., 2013).

#### Experiences in North America

Wheat is an important rotational crop, often grown in Canada as the dominant cereal in crop rotations with other commodities including pulses and oilseeds. Pulses like field pea (*Pisum sativum* L.) or lentil (*Lens culinaris* Medic.) are sensitive to many herbicides, leaving limited options for chemical weed control. Many of the herbicides that are effective in these crops (e.g., the ALS inhibitors) have been rendered ineffective on several weed species with evolved resistance to active ingredients within this herbicide mode-of-action. Alberta and Saskatchewan, Canada, alone is home to 18 weed species with confirmed resistance to ALS-inhibiting herbicides (Beckie et al., 2017; Heap, 2019). The cereal phase in the crop rotation provides an excellent opportunity to manage ALS inhibitor-resistant weeds and facilitate inclusion of high-value pulses in crop rotations. In durum, post-emergent application of synthetic auxins (group O/4), photosystem II inhibitors (nitriles) (group C3/6), or (4-hydroxyphenylpyruvate dioxygenase) HPPD inhibitors (group F2/27) can be an excellent rotational weed management strategy, however re-cropping restrictions must be considered when using some of these active ingredients (Geddes et al., 2018). Durum wheat is unique from common wheat as it can be more sensitive to certain herbicides such as pyroxasulfone (Soltani et al., 2012).

Optimizing plant spatial arrangement can help improve the ability for durum to compete with weeds. Some reports indicate that narrower row spacing (5 cm vs. 15 cm vs. 25 cm) improves the weed competitive ability of durum over increased seeding densities (De Vita et al., 2017). Moreover, row widths >25 cm reduced yield of wheat. The greater impact of row spacing compared with seeding density is likely due to reduced tillering in durum compared with bread wheat cultivars, which could limit the extent to which durum occupies inter-row niche space compared with bread wheat classes. However, increased durum seeding rates may be a beneficial management tool in zero- or minimum-tillage production systems, where moderate (20–30 cm) row spacings are commonplace. Indeed, increased durum seeding densities are correlated positively with increased leaf area index (Isidro-Sánchez et al., 2017), which can hasten crop canopy closure and reduce the quality of light available to weeds beneath the crop canopy. Optimal plant spatial arrangement by reducing row spacing and using high durum seeding densities (≥400 seeds m^–2^) can improve the ability for durum to compete with weeds and thus reduce selection pressure for herbicide resistance in durum production.

Recent advances in multispectral or hyperspectral imaging technologies hold promise for real-time and site-specific weed management. Several problematic weeds in durum, including wild oat and annual ryegrass, can be successfully discriminated from durum wheat using multispectral imaging in the 400–900 nm range (Lopez-Granados et al., 2008). These methods hold promise for mapping of weed patches in durum, allowing for cost-effective herbicide application including additional effective sites-of-action on herbicide-resistant weed patches.

According to weed surveys of Alberta and Saskatchewan, Canada conducted between 2009 and 2017, the most abundant weeds found in durum wheat were green foxtail, wild oat, volunteer canola [*Brassica napus* L.), stinkweed (*Thlaspi arvense* L.), and wild buckwheat (*Fallopia convolvulus* (L.) Á. Löve] (Geddes et al., 2018). Within these weeds, green foxtail, wild oat, and wild buckwheat have been known as the most abundant weeds in all crops in the prairies since the 1970s. Both green foxtail and wild oat are spring annuals that cause yield loss in durum depending on emergence timing of the weeds and weather conditions (Douglas et al., 1985; Beckie et al., 2012). Green foxtail exhibits seed dormancy at maturity for approximately 10 weeks and wild oat exhibits it for almost one year, resulting in moderately persistent seed banks. Green foxtail is highly responsive to nitrogen fertilizer and can be managed using suitable fertilization techniques like mid- or side-row banding. Volunteer canola is an annual weed, with seeds in the soil seed bank maintaining viability for about three years. Presence of this weed is often caused by large seed losses at canola harvest, resulting in glyphosate, glufosinate, or imidazolinone resistant populations in subsequent crops (Gulden et al., 2008). Disturbing soil shortly after canola harvest can reduce the density of volunteer canola originating from crop harvest losses (Geddes and Gulden, 2017). Wild buckwheat is a spring annual weed with seeds germinating within the first year in the seed bank, and surviving up to five years. It can cause significant crop lodging, grain sample contamination, and harvest difficulties. It has the ability to climb the canopy to capture light (Hume et al., 1983). Stinkweed has the ability to grow like a spring or winter annual, with viability for over 20 years in the soil and causing yield losses in many crops (Warwick et al., 2002).

#### Experiences in Australia

The evolution of herbicide resistance highlights the need for alternative forms of weed control including improving the competitiveness of the durum crop. The main aim is to increase the competitive ability of the crop against weed species, this not only means lower yield losses from weed competition but also greater suppression of weed growth and seed production (Bajwa et al., 2017). Agronomic or genetic intervention to improve crop competition works particularly well when the weed and crop are phenotypically similar, such as in the case of ryegrass and wheat (Lemerle et al., 2001).

Durum has typically reduced early vigor and crop competition with weeds when compared to other cereal crop options in Australia. A comprehensive study of bread wheat and durum wheat genotypes from all over the world found significant variation in the competitiveness of genotypes on annual ryegrass (Lemerle et al., 1996). Overall, bread wheat was more competitive against ryegrass than durum wheat. In Australia, there has also been improvements in plant breeding for crop vigor in durum, the introduction of new durum cultivar DBA Aurora has demonstrated a similar ability to reduce ryegrass seed set through competition when compared to bread wheat (Goss and Wheeler, 2014).

**FIGURE 6:**
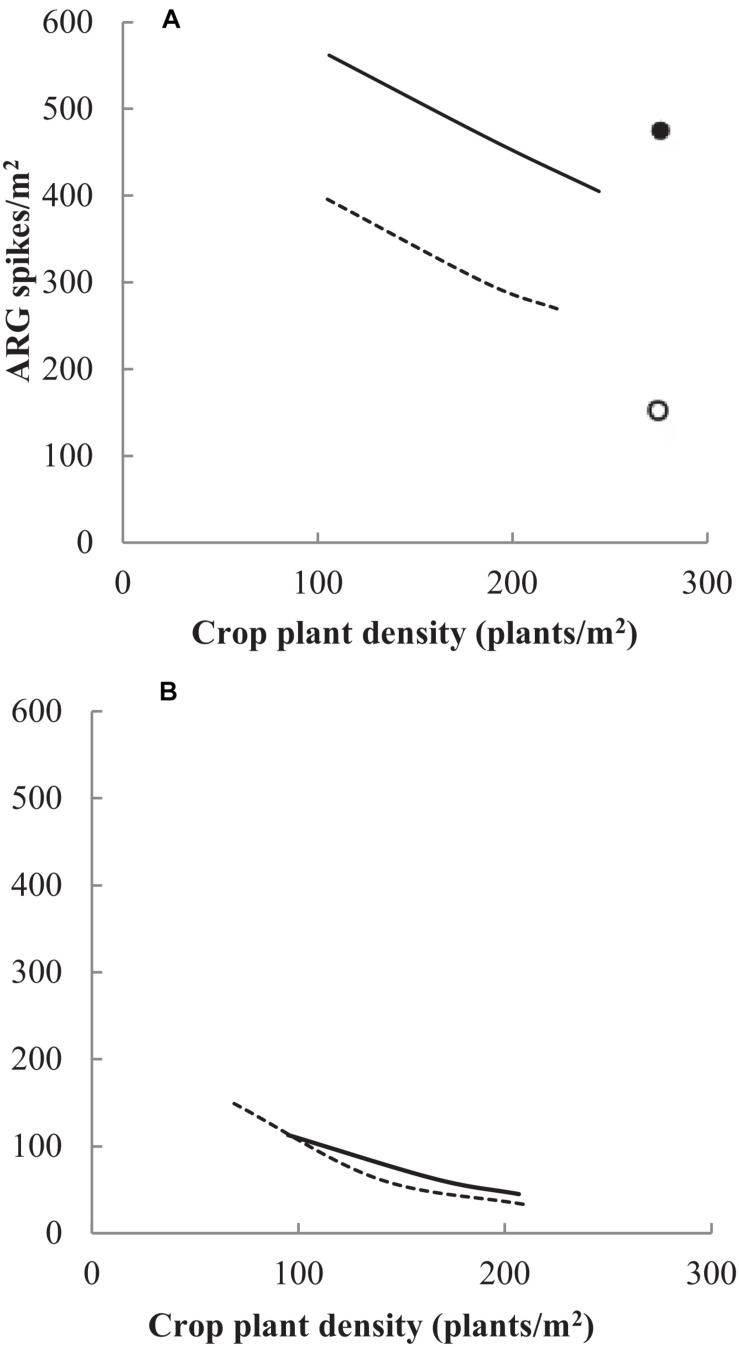
**(A)** No pre-emergent herbicide and **(B)** treated with pre-emergent herbicide. The effect of the management combination of genotype, herbicide management, and plant density on the survival and density of annual ryegrass spikes (ARG) at Tarlee 2012 (LSD 5% = 55 spikes/m^2^). Two durum varieties Saintly (Ο) and Yawa (•) in combination without a pre-emergent herbicide treatment (A) and treated with the pre-emergent herbicide BoxerGold at 2.5 L/ha incorporated by sowing (B) at three crop plant density levels. Original work.

To encourage integrated G × E × M approaches in South Australia, Porker and Wheeler (2012) demonstrated that the management combination of variety, seeding rate, and herbicide all play a significant role in the success in managing annual ryegrass (Figure 6). The pre-emergent herbicide combination of prosulfocarb plus S-metolachlor remains the main factor providing the greatest proportion of weed control. However combining this with more vigorous varieties such as Saintly and increasing seeding rates from 100 to 300 seeds m^–2^ resulted in increased yield, improved weed competition and reduced weed seed set (Figure 6). This is consistent with bread wheat examples and the recommendation for higher wheat seed rates as part of an integrated weed management strategy is now strongly promoted to farmers (Lemerle et al., 2004; Bajwa et al., 2017).

Sowing time also plays a key role in weed control. The benefit of earlier sowing across most of the southeast Australian wheat belt is now well-established (Flohr et al., 2018; Hunt et al., 2019b). It is often tempting for growers to delay sowing in order to achieve greater control of weeds with additional tillage; however, these practices have been shown to significantly contribute to the yield gap in Australian wheat (Hochman and Horan, 2018). Sowing earlier in warmer soils also enables wheat a competitive advantage against weeds (Gomez-Macpherson and Richards, 1995) and, when combined with effective pre-emergent herbicides, can provide adequate control of annual ryegrass and reduced seed production without compromising yield (Preston et al., 2017). Broader adoption of alternative, non-chemical management options in-conjunction with herbicide weed management programs is essential for sustainable durum crop production in Australia.

## Conclusion

Globally, durum is still considered a minor wheat crop and typically much of the research effort on durum often is conducted in conjunction with studies of bread wheat. Moreover, management practices are often borrowed from research conducted on common wheat. While we have sought to present synergies possible with a G × E × M paradigm, it is clear a coordinated effort and cross-disciplinary approach is yet to be fully realized, which underscores the need for transformational research that exemplifies the G × E × M paradigm (Beres et al., 2020). In many areas of the world, climate change will drive future research and innovation, which requires a re-think of how we link together all the genetic and management components when designing a resilient cropping system (Fletcher et al., 2020). Rather than commonplace ‘reactive agronomy’, transformational research will be needed where there is greater emphasis on integration and optimization of the overall G × E × M system by the agronomist (Hunt et al., 2019a). Agronomists must be the leaders, the translators and the communicators, accessing the best discipline-based knowledge and expertise where relevant to deliver transformational change. Kirkegaard and Hunt (2010) demonstrated how a systems approach can transform a wheat-based cropping system averaging 1.6 Mg ha^–1^ with inherent sustainability issues into a resilient system that increased attainable yield by 3×. This represents the type of modified paradigm needed to address the very real threat of declining rates of yield growth (Fischer and Connor, 2018). A major component when defining resiliency will be the need for systems to be sustainable. Moreover, the sustainable intensification of cropping systems is considered the only feasible path to meet future food demands (durum no exception) as it is recognized that further conversions of natural ecosystems to farmland is not tenable (Cassman and Grassini, 2020). The Wheat Initiative^4^ could provide the framework in charting this path forward as it brings together wheat experts from around the world to participate in cross-disciplinary teams and working groups to establish priorities for global wheat research.

Designing robust agronomic systems for durum demands scientific creativity and foresight based on a deep understanding of constitutive components and their innumerable interactions with each other and the environment. Advances in individual technologies (e.g., genetic improvements, new pesticides, seeding technologies) are of little benefit until they are melded creatively and thoughtfully into resilient Genotype × Environment × Management (G × E × M) systems that will flourish in the field under unpredictable conditions of prairie farmlands.

## Author Contributions

BB and ER prepared and edited the original manuscript with input, revisions, and editorial contributions provided by all co-authors. JC, CG, PG, KP, and JR provided major contributions to the sections. All authors contributed to the article and approved the submitted version.

## Conflict of Interest

The authors declare that the research was conducted in the absence of any commercial or financial relationships that could be construed as a potential conflict of interest. The reviewer HK declared a shared affiliation, with no collaboration, with several of the authors, JC and CP, to the handling editor at the time of review.

## Footnotes

1 www.yieldgap.org

2 https://www.wheatinitiative.org

3 http://www.10wheatgenomes.com/

4 www.wheatinitiative.org
